# FACTORS ASSOCIATED WITH CAREGIVER REACTIONS TO PROBLEM BEHAVIORS OF PERSONS WITH ALZHEIMER’S DISEASE

**DOI:** 10.1093/geroni/igz038.1057

**Published:** 2019-11-08

**Authors:** Junrong Shi, Cathy Scott

**Affiliations:** University of Tennessee at Chattanooga, Chattanooga, Tennessee, United States

## Abstract

Problem behaviors among persons with Alzheimer’s Disease (AD) can have a major impact on caregivers. However, caregiver’s subjective reactions to the problem behaviors have a stronger impact on caregivers than the objective frequency of problem behaviors (Robinson et al., 2001). This study aims to examine the factors associated with caregiver’s subjective reactions to problem behaviors. Data were collected from a sample recruited from community agencies served AD caregivers in the southeastern region of the country (N=109). The caregivers’ reactions to problem behaviors were measured by the Revised Memory and Behavior Problem Checklist (Teri et al., 1992). Three subscale scores were used to measure the reactions to the behaviors related to memory loss, depression and disruption. Multivariate regression models were conducted including gender, race, employment, living arrangement, knowledge about the disease, resilience for caregivers; and ADL and IADL functioning, and frequency of problem behaviors for care recipients. Race (B=-.162; p<.05) and frequency of care-recipient problem behaviors (B= 0.733; p<.001) were significantly associated with caregiver’s reaction to problem behaviors. Caregiver’s knowledge about the disease (p<.01) only influence their reactions to memory loss problems but not for disruption and depression problems. African American caregivers had fewer reactions to disruption (p<.01) and more reactions to depression problems (p=0.06) than white caregivers, but no difference between the two groups in their reactions to memory loss problems. Personal resilience was not associated with reactions to any problem behaviors. Intervention should be tailored to the needs of caregivers to deal with behaviors of a person with AD.

